# *Syzygium cumini* Nectar Supplementation Reduced Biomarkers of Oxidative Stress, Muscle Damage, and Improved Psychological Response in Highly Trained Young Handball Players

**DOI:** 10.3389/fphys.2018.01508

**Published:** 2018-10-31

**Authors:** Layanna Cibelle de Sousa Assunção Carvalho, Marcelo Conrado de Freitas, Alexandre Sergio Silva, Aline Camarão Telles Biasoto, Maria do Carmo de Carvalho e Martins, Rayane Carvalho de Moura, Ana Karolinne da Silva Brito, Acacio Salvador Veras e Silva, Sérgio Luiz Galan Ribeiro, Fabrício Eduardo Rossi, Marcos Antônio Pereira dos Santos

**Affiliations:** ^1^Department of Nutrition, Federal University of Piauí, Teresina, Brazil; ^2^Skeletal Muscle Assessment Laboratory, Department of Physical Education, School of Technology and Sciences, São Paulo State University, São Paulo, Brazil; ^3^Department of Nutrition, University of Western São Paulo, Presidente Prudente, Brazil; ^4^Laboratory Study of Physical Training Applied to Performance and Health, Federal University of Paraiba, João Pessoa, Brazil; ^5^Brazilian Agricultural Research Corporation, Federal Institute of Piauí, Piauí, Brazil; ^6^Group of Studies on Exercise Physiology Applied to Performance and Health, Department of Biophysics and Physiology, Federal University of Piauí, Teresina, Brazil; ^7^Immunometabolism of Skeletal Muscle and Exercise Research Group, Department of Physical Education, Federal University of Piauí, Teresina, Brazil

**Keywords:** performance, nutrition, sport, athletes, recovery

## Abstract

The purpose of this study was to investigate the effects of *Syzygium cumini* (SC) nectar supplementation on performance, markers of oxidative stress, muscle damage, and psychological response in Handball players. Twenty-five young athletes (age = 18.6 ± 2.4 years) from an elite high school national level Brazilian Handball team were randomized into two study groups: SC/Jamelon nectar (SC, *n* = 12) and placebo (*n* = 13). The subjects ingested 10 mL/kg/day of Jamelon nectar or placebo 30 min before the training sessions and immediately after training cessation, for 28 days. Body mass index (BMI) and percentage of fat mass were assessed using bioelectrical impedance analysis. Biomarkers of oxidative stress were measured by lipid peroxidation, which was quantified by malondialdehyde (MDA). Total antioxidant capacity (TAC), creatine kinase (CK) activity, and lactate dehydrogenase (LDH) were determined. The 20 m shuttle run test, vertical jump, and running anaerobic sprint test were assessed to verify performance and the fatigue index was calculated. The Profile of Mood States (POMS) questionnaire was used for psychological evaluation. Both groups demonstrated improved vertical jump performance and a decreased fatigue index over time but without significant differences between them regarding performance. There was statistically significance only for SC in CK, LDH, and MDA, and TAC was greater in the SC compared to placebo. Furthermore, only the SC group demonstrated improved mood disturbance and confusion after the intervention. In conclusion, the present study suggests that SC nectar supplementation reduced biomarkers of oxidative stress and muscle damage, and improved psychological response in young handball players.

## Introduction

Handball is a sport modality characterized by performing many different movements with intensive effort, such as running, jumping, changing direction, shooting, and technical movements (Michalsik et al., 2015). In addition, handball players are involved in repeated bouts of training and games with high physical contact, increasing the mental and physical demand on the players (Michalsik et al., 2014). Excessive training can generate imbalance in the pro and antioxidant status due to the inefficient ability of the antioxidant system to reduce overproduction of reactive oxygen species (ROS), thus this condition may lead to oxidative stress (Hattori et al., 2009; Martarelli and Pompei, 2009; Mrakic-Sposta et al., 2015). Marin et al. (2013) investigated the response of oxidative stress and antioxidant status in handball players for 6 months with different training loads and competitions. The authors observed an increase in antioxidant capacity and biomarkers of oxidative stress (superoxide anion and hydrogen peroxide) according to training load.

In addition, Tanskanen et al. (2010) demonstrated an increase in the resting biomarker of oxidative stress (protein carbonyls) in over-trained athletes; however, there was a significant relationship between over-training and low antioxidant capacity, demonstrating that excessive training plus insufficient recovery periods can induce overproduction of ROS (Marin et al., 2013). Therefore, oxidative stress plays a role in the development of overtraining syndrome, which can be associated with muscle damage, inflammation and reductions in psychological aspects (e.g., motivation and mood) as well as performance in athletes (Purvis et al., 2010; Tanskanen et al., 2010; Carfagno and Hendrix, 2014). In this perspective, other studies show association between oxidative stress with muscular fatigue, indicating that oxidative stress during exercise decreases the skeletal muscle contractility by impairing the release of calcium by the sarcoplasmic reticulum (Moopanar and Allen, 2005; Cheng et al., 2016), which could result in a lower interaction between actin-myosin filaments and force output (Powers et al., 2011).

For this reason, studies have investigated the influence of different type of dietary antioxidants on performance and oxidative stress in athletes, demonstrating an efficient strategy to counter high ROS production in this population (Peternelj and Coombes, 2011; Pingitore et al., 2015). *Syzygium cumini* (SC), commonly known as Jambolan, is used for the treatment of several diseases including diabetes, inflammation, sore throat, bronchitis, asthma, coughs, and dysentery (Ayyanar and Subash-Babu, 2012), as the health benefits of SC consumption have been associated with high phenolic compounds, such as gallic acid and flavonoids (catechin, epicatechin, epigallocatechingallate, and epicatechingallate; Ayyanar and Subash-Babu, 2012).

Investigation of the health effects of SC supplementation in animals is widely explored in the literature, demonstrating antioxidant and anti-inflammatory activity, as well as hypoglycemic effects (Ravi et al., 2004; Sharma et al., 2008; Siani et al., 2013). Ulla et al. (2017) showed that SC seed powder prevents oxidative stress in rats with high fat diet induced obesity through a decrease in malondialdehyde (MDA) levels and increased activity of antioxidant enzymes, superoxide dismutase, glutathione peroxidase, and catalase. Whether SC supplementation influences exercise performance, oxidative stress, muscle damage, and psychological response in humans is currently unknown in the literature. We hypothesized that SC supplementation could reduce oxidative stress, muscle damage, and improves performance as well as psychological response in handball players.

Thus, the purpose of this study was to investigate the effects of SC nectar supplementation on performance, markers of oxidative stress and muscle damage, and psychological response in handball players.

## Materials and Methods

### Subjects

The study was carried out at the Federal University of Piauí (UFPI), Teresina, Brazil. Twenty-five young male athletes, age between 16 and 23 years (*n* = 25; age = 18.6 ± 2.4 years) from the elite high school national level handball team (CaicBalduíno school/GHC team and positions of the players: 13 center, 9 wing, and 3 pivot) were included, who had been training for at least 1 year with a weekly frequency of five training sessions,90–120 min per day, and had regularly participated in international and national competitions during the previous 2 years. The team is currently twice world champion and national champion in the category.

The athletes were randomized into two study groups: SC (*n* = 12) and placebo (*n* = 13), using simple randomization techniques for allocation, which ensure that each athlete has an equal chance of being allocated to a treatment group. None of the participants reported any physical limitations that could prevent completion of the assessments and exercise interventions; being smokers or continuously using any medication, in addition to which, none were taking any dietary or performance enhancing supplements that could have affected the outcome of the study. During the study, the participants were required not to consume nectar or derivatives other than the intervention. The project was approved by the Ethics Research Group of the UFPI, Teresina, Brazil (Protocol Number: 1755888) and the research was conducted according to the 2008 Revision of the Declaration of Helsinki. All participants and their parents signed a written consent form and were informed about the purpose of the study and the possible risks.

### Experimental Design

This study used a randomized, double-blind design. Initially, the participants were submitted to body composition, dietary intake, and psychometric assessments, and collection of blood samples; 24 h later, they performed aerobic, anaerobic, and strength tests. Subsequently, the participants were randomized, using simple randomization^1^, into two groups (SC or Placebo) and after 28 days of supplementation the same initial evaluation was performed. After intervention (48 h), blood samples were collected and performance tests were conducted after 72 h.

### Procedures

#### Training Program and Supplementation Protocol

The training program following a model proposed by Verjoshanski (1990) and adapted by Oliveira (1998), which we used Phase A1 (4 weeks) of periodization, according to Souza et al. (2006). During 28 days of intervention, the coach maintained the same training session, which the athletes performed general strength and medium-intensity endurance training about 60 min in the morning, three times a week. Twice a week, the subjects trained maximal power and maximal speed and 5 days a week in the evening (6 to 7:30 p.m.) trained sport-specific strength and power and techno-tactical skills. Participants were instructed to maintaining heart rate (HR) between 75–90% of maximum HR and the rating of perceived exertion between 6 and 9 (Foster et al., 2001).

The individuals consumed 10 mL kg/day of Jamelon nectar, divided into the moments before the training session (30–45 min pre-training) and immediately after the training session, for 28 days, according to the protocol adopted by Toscano et al. (2015). On days when there was no training, supplementation was consumed with snacks, at the time of choice of the athletes.

For supplementation, a Jamelon nectar drink was produced with 100 g of filtered drinking water, 4 g of crystal sugar (Cristal^®^), and 33.3 g pulp (edible fraction with skin). In the processing of the nectar, the Jamelon pulp was thawed at 25°C and homogenized with sugar and water in an industrial blender (Siemsen, D560484, Jaraguá do Sul, Brazil). Each nectar drink was poured by hand into a plastic bottle with a capacity of 1 L, previously sanitized in hypochlorite solution, and capped with a plastic lid. The bottles containing the nectar drink were stored in a refrigerator at 5°C.

The phenolic profile of the Jamelon nectar used in the present study was evaluated by the Embrapa Semi-Arid Oenology Laboratory (Petrolina, Pernambuco, Brazil) using a validated and internationally published procedure (Kschonsek et al., 2018). Twelve phenolic compounds were positively identified and quantified by HPLC using an Aliance Waters 2695 system (Milford, MA, United States) equipped with a diode array detector (DAD) and fluorescence detector (FLD). Data acquisition and analysis were performed with Waters Empower^TM^ 2 software (Milford, MA, United States).

The control group received a carbohydrate beverage that was administered in isocaloric, isoglucose, and isovolumetric form, by means of a conversion chart according to the weight of each athlete, as proposed in the study by Toscano et al. (2015) who used carbohydrate drink as a control for fruit juices. The nutritional composition of the two beverages is shown in Table 1.

**Table 1:** Nutritional composition and HPLC-evaluated phenolic compounds of the supplemented beverages.

Nutritional composition	Nectar of Jamelon (33 g of pulp + 4 g of sugar + + 100 mL of water)	Drink control (8 g of maltodextrin 100 mL of water) 100 mL of water)
Calories (kcal)	30	30
Carbohydrates (g)	7.5	7.5
Proteins (g)	0.17	0
Lipids (g)	0.03	0
Fibers (g)	0.6	0
Gallic acid	2.61 ± 0.09	–
Flavonoids (mg/L)	0.61 ± 0.00	–
(+) – Catechin	0.53 ± 0.00	–
(−) – Epicatechin	1.39 ± 0.07	–
(−) – Epicatechin gallate	2.12 ± 0.05	–
(−) – Epigallocatechin gallate	0.74 ± 0.00	–
Procyanidin A2	0.71 ± 0.00	–
Procyanidin B1	0.46 ± 0.00	–
Procyanidin B2		
Anthocyanins (mg/L)	7.53 ± 0.16	–
Cyanidin-3.5-di-*O*-glycoside	1.44 ± 0.13	–
Cyanidin-3-*O*-glycoside	205.7 ± 2.51	–
Malvidine-3.5-di-*O*-glycoside	48.85 ± 1.08	–

*Data are mean ± standard deviation.*

#### Anthropometric, Body Composition, and Dietary Intake Assessment

Body weight was measured using an electronic scale (Filizzola PL 50, Filizzola Ltda., São Paulo, Brazil), with a precision of 0.1 kg. Height was measured on a fixed stadiometer of the brand Sanny (Sanny brand, São Paulo, Brazil) with an accuracy of 0.1 cm and length of 2.20 m. The body mass index (BMI) was calculated as body mass divided by the square of the body height (kg/m^2^). The percentage of fat mass was assessed using bioelectrical impedance analysis and accompanying software (OMRON^®^ BIA, model 214, OMRON HEALTHCARE Co., Ltda., Kyoto, Japan) and the athletes were positioned in a supine position and remained still throughout the examination and they were wearing light clothing. Twenty-four daily records were conducted via 3-day food diaries that consisted of one weekend day, Sunday, and two weekdays, Monday and Wednesday or Tuesday and Thursday. All food intake records were analyzed for total kilocalorie and macronutrient intakes to ensure that dietary intake was similar between baseline and post-intervention. Food questionnaires were analyzed by the same nutritionist using the software NutWin, version 1.5 (Nutrition Support Program, Federal University of São Paulo, São Paulo, Brazil, 2002).

#### Biomarkers of Oxidative Stress

After an overnight fast (12 h), venous blood samples were collected, which it was 24 h before the beginning of the intervention and 48 h after 28 days of supplementation. Blood samples (10 mL) were immediately allocated into two 5 mL vacutainer tubes (Becton Dickinson, Juiz de Fora, Brazil) containing EDTA for plasma separation. The tubes were centrifuged at 3000 rpm for 15 min at 4°C, and plasma and serum samples were stored at −20°C until analysis. Biomarkers of oxidative stress were measured by lipid peroxidation, which was quantified by MDA. The thiobarbituric acid reaction in plasma was adopted according to the method described by Ohkawa et al. (1979). In addition, total antioxidant capacity (TAC) was determined. TAC was quantified in plasma by measuring the activity of elimination of the 2,2-diphenyl-1-picrylhydrazyl free radical using the method described by Brand-Williams et al. (1995). The absorbance was obtained in the Labmax 240 premium automatic analyzer (Labtest, Minas Gerais, Brazil), at 520 nm wavelength.

#### Muscle Damage

The plasma creatine kinase (CK) activity and plasma lactate dehydrogenase (LDH) were determined 48 h after the supplementation period, after an overnight fast (12 h) using the catalytic activity method and pyruvate lactate method, respectively, both with a commercial Kit (Labtest, Minas Gerais, Brazil) and were quantified using Lab-Max 240 Premium (Labtest, Minas Gerais, Brazil) according to the manufacturer’s instructions.

### Performance

#### Lower Body Power

Vertical jump was assessed using the Jump test on the Mat (Multi-Sprint^®^ software, Huntsville, AL, United States). Subjects were instructed to stand on the mat with feet hip width apart and perform a rapid lower body eccentric movement followed immediately by a maximal intensity concentric movement. Subjects were instructed to jump straight up and minimize any in-air hip flexion. The best of three trials separated by a 1 min rest interval was recorded as vertical jump height (cm), according to Hermassi et al. (2011) and Silva et al. (2016). The lower body power test was performed immediately post body composition assessment at baseline and after 28 days.

#### Cardiorespiratory Fitness

The 20 m shuttle run test (20MST) was used to determine maximum oxygen consumption (VO_2_max). The test was performed incrementally at a distance of 20 m back and forth. The VO_2_max was determined by the following equation: age < 18 years: VO_2_max = –24,4 + (6^∗^Vmax) and age > 18 years: VO_2_max = 31.025 + (3.238^∗^Vmax) – (3.248^∗^age) + (0.1536^∗^Vmax^∗^age); where Vmax is the speed (km/h^−1^) obtained in the final completed stage (Leger et al., 1988).

After 24 h of 20MST, anaerobic capacity was evaluated using the running anaerobic sprint test (RAST; Silva et al., 2016). After a pre-warm-up by stretching and light jogging for 10 min and an active recovery of 5 min, athletes performed six full-length races over a distance of 35 m at the maximum possible speed, with a 10 s rest interval between each repetition. The times in seconds and hundredths of a second and the speed in m/s were recorded using Hidrofit^®^ brand photocells and Multi-Sprint^®^ software.

The anaerobic power of each sprint in W/kg was obtained by multiplying the current weight of the athlete by the distance squared and then dividing by the time obtained in the race cubed. This power output (W) was then divided by the current weight (kg) of the individual, obtaining the maximum power in W.kg^−1^ (Bangsbo, 1998). All performance tests were conducted 72 h after 28 days of supplementation and the participants were instructed to avoid strenuous physical exercise and had not used any caffeinated beverages for at least 24 h prior tests.

#### Psychological Evaluation

In order to evaluate the initial state of mood of the athletes, associated with psychological stress, the subjects responded to the version of the Profile of Mood States (POMS) questionnaire adapted to the sport by Raglin and Morgan (1994) at the beginning and after the 28-day intervention. This questionnaire is composed of 36 items, distributed in six dimensions – tension, depression, hostility, fatigue, confusion, and stamina.

### Statistical Analysis

We performed a power analysis of this study based on the observation from a previous study that verified a difference of 0.2 for VO_2max_ (mL.kg^−1^⋅min^−1^) with a standard deviation of 0.2 in the experimental and control means after 28 days of integral purple grape juice on the performance of recreational runners (Toscano et al., 2015). Using a power (1-type II error) of 0.80 and a type I error of 0.05 by PS software (ver 3.1.2, Dupont and Plummer^2^), it was estimated that we would need 17 participants per group. Therefore, we were able to reject the null hypothesis that this response difference is zero with a power (1-type II error) of approximately 70%.

A mixed model was used to compare placebo and SC nectar across time using a two-way ANOVA for repeated measures. When a significant interaction (group x time) was observed, a Bonferroni *post hoc* test was conducted. For all measured variables, the estimated sphericity was verified according to the Mauchly’s W test and the Greenhouse–Geisser correction was used when necessary. The partial eta-squared (η^2^) was calculated for time. Statistical significance was set at *p* < 0.05. The data were analyzed using StatSoft Statistica (version 10.0).

## Results

Table 2 presents the dietary and macronutrient intake, anthropometry, body composition, and performance variables at baseline and after 28 days of intervention in the placebo and SC groups. There were no statistically significant differences between groups at baseline and across time for any variable investigated.

**Table 2:** Comparison between placebo and supplementation group on dietary intake, anthropometry and performance.

Variables	Placebo (*n* = 13)		SC (*n* = 12)	
	Baseline	Post	Baseline	Post
CHO (g)	900.7 ± 413.1	1153 ± 545.8	1041 ± 341.7	1430 ± 665.1
PRO (g)	461.9 ± 172.5	404.5 ± 204.2	404.2 ± 129	486.1 ± 188.2
FAT (g)	590.3 ± 388.5	613.3 ± 295.7	702 ± 247.9	869.8 ± 389.8
Total Intake (kcal)	1963 ± 775	2265 ± 865	2116 ± 732	2562 ± 913
Vitamin A (μg ER)	346.5 ± 266.9	311.6 ± 184.9	280.9 ± 127.9	337.4 ± 256.6
Vitamin C (mg)	21.8 ± 16.6	23.4 ± 18.6	30.9 ± 7.7	36.4 ± 16.3
Vitamin E (mg)	13.1 ± 6.7	19.2 ± 9.2	12.2 ± 7.8	14.6 ± 3.3
Copper (mg)	0.9 ± 0.5	1.1 ± 0.4	0.9 ± 0.2	1.3 ± 0.5
Iron (mg)	13.2 ± 6.1	16.8 ± 5.7	14.9 ± 3.2	20.5 ± 9.0
Zinc (mg)	11.3 ± 7.2	9.8 ± 3.2	12.5 ± 3.4	13.9 ± 7.4
Selenium (μg)	102.9 ± 44.8	106.6 ± 39.5	108.3 ± 24.4	99.9 ± 36.9
Body mass (kg)	71.2 ± 7.1	71.9 ± 7.1	70.7 ± 7.6	70.8 ± 7.9
BMI (kg/m^2^)	22.1 ± 1.8	22.1 ± 1.7	22.2 ± 2.6	22.3 ± 2.5
Body fat (%)	18.0 ± 3.5	17.9 ± 3.5	18.1 ± 4.5	18.1 ± 4.6
VO2_max_ (mL⋅kg^−1^⋅min^−1^)	45.7 ± 3.8	46.5 ± 5.4	48.3 ± 6.3	49.1 ± 6.9
RAST (W/kg^−1^)	8.6 ± 1.2	8.7 ± 1.2	7.8 ± 1.3	8.1 ± 1.1
Fatigue index (W⋅seg^−1^)	8.1 ± 2.4	6.8 ± 2.7^¥^	6.7 ± 2.3	5.7 ± 1.9^¥^
Vertical Jump (cm)	36.2 ± 5.6	37.2 ± 5.2^¥^	35.8 ± 3.3	36.3 ± 3.2^¥^

*^¥^Main effect of time with significant difference from baseline. VO_2max_, maximal oxygen consumption; RAST, running anaerobic sprint test.*

### Performance

For vertical jump, both groups improved over time (*F* = 4.641, *p* = 0.042, η^2^ = 0.17), with no significant differences in changes between groups (*F* = 0.117, *p* = 0.736) and interaction (*F* = 0.587, *p* = 0.451). For fatigue index, there was a main effect of time (*F* = 8.668, *p* = 0.008, η^2^ = 0.30) but no difference between groups and interaction was observed (*p* > 0.05). For VO_2_max and RAST, there were no main effects of time, statistically significant interactions, or differences between groups (*p >* 0.05).

### Biomarkers of Oxidative Stress and Muscle Damage

Table 3 shows the comparison between placebo and supplementation groups for muscle damage and oxidative stress.

**Table 3:** Comparison between placebo and supplementation groups on muscle damage and oxidative stress.

Variables	Placebo (*n* = 13)		SC (*n* = 12)	
	Baseline	Post	Baseline	Post
CK (U/L)	179.6 ± 52.2	186.1 ± 60.5	210.2 ± 65.34	150.2 ± 58.2*
LDH (U/L)	292.7 ± 45.3	296.8 ± 55.8	331.9 ± 52.4	288.9 ± 37.4*
MDA (μM)	4.0 ± 1.0	4.3 ± 0.7	4.6 ± 1.1	3.2 ± 0.9*
TAC (%)	17.2 ± 2.8	17.7 ± 2.3	18.3 ± 3.1	21.8 ± 2.7*^$^

*Data are mean ± standard deviation. ^∗^Bonferroni’s post hoc with statistic significant difference from baseline; ^$^statistic significant difference between groups. CK, Creatine kinase; LDH, lactate dehydrogenase; MDA, malondialdehyde; TAC, total antioxidant capacity.*

For CK, there was a statistically significant interaction (*F* = 5.981, *p* = 0.023) with lower CK concentration in the SC group after intervention; however, there was no significant difference between groups (*F* = 0.010, *p* = 0.891). For LDH, there was a statistically significant interaction (*F* = 4.365, *p* = 0.048). The Bonferroni *post hoc* showed a decrease in the SC group after 28 days of supplementation but no change was observed in the placebo.

For MDA, there was a main effect of time (*F* = 5.865, *p* = 0.024, η^2^ = 0.20) and a statistically significant interaction was observed (*F* = 15.847, *p* = 0.001). The *post hoc* test demonstrated that only the SC group presented decreased MDA in relation to baseline but there was no significant difference between groups (*F* = 0.713, *p* = 0.407).

For TAC, there was a main effect of time (*F* = 19.378, *p <* 0.001, η^2^ = 0.46), statistically significant interaction (*F* = 11.400, *p* = 0.003), and significant difference between groups (*F* = 6.820, *p* = 0.016). The Bonferroni *post hoc* showed a greater mean of CAT in the SC group than the placebo (*p* = 0.004).

### Psychological Response

Table 4 presents the difference between placebo and supplementation groups for the psychological response.

**Table 4:** Comparison between placebo and supplementation groups on the psychological response.

Variables	Placebo (*n* = 13)		SC (*n* = 12)	
	Baseline	Post	Baseline	Post
Mood disturbance	99.9 ± 12.4	97.7 ± 12.7	100.7 ± 17.0	89.7 ± 11.6*
Confusion	−1.5 ± 2.1	−1.6 ± 1.8	−1.7 ± 2.6	−3.8 ± 2.1*
Tension	−0.31 ± 2.1	−0.46 ± 2.0	0.25 ± 2.9	−1.1 ± 1.7
Depression	5.1 ± 4.6	4.8 ± 5.0	3.9 ± 7.8	2.2 ± 1.6
Hostility	5.1 ± 2.1	5.0 ± 2.4	4.2 ± 4.3	3.2 ± 2.3
Vigor	13.8 ± 6.8	14.1 ± 5.6	12.0 ± 6.7	15.8 ± 4.7
Fatigue	5.1 ± 3.3	4.1 ± 2.6	6.2 ± 5.2	4.9 ± 3.7

*Data are mean ± standard deviation. ^∗^Bonferroni’s post hoc with statistic significant difference from baseline.*

For mood disturbance, there was a main effect of time (*F* = 10.509, *p* = 0.004, η^2^ = 0.31) and statistically significant interaction (*F* = 5.336, *p* = 0.030). The *post hoc* only identified an improvement in the SC group across time (*p* = 0.004), however, without differences between groups. Regarding confusion, there was a main effect of time (*F* = 11.923, *p* = 0.002, η^2^ = 0.34) and statistically significant interaction (*F* = 10.344, *p* = 0.004) with a decrease in the SC group but not the placebo; however, there was no significant difference between groups (*F* = 2.120, *p* = 0.159).

Regarding correlations, there was a statistically significant relationship between absolute changes in the CK and mood disturbance only for the SC group (*r* = 0.74, *p* = 0.006) but no correlation was observed for the placebo (*r* = 0.06, *p* = 0.833) and a tendency to significant correlation was observed between CK-delta and confusion-delta in the SC group (*r* = 0.55; *p* = 0.06) but not in the placebo (*r* = 0.13; *p* = 0.67).

## Discussion

The main findings of this study were that SC nectar decreased muscle damage markers (CK and LDH) and MDA after 28 days of intervention in handball players and increased TAC. Furthermore, SC nectar improved psychological response with lower mood disturbance compared to baseline and a significant relationship was observed between changes in the CK concentration and mood disturbance only in the SC group.

It has been reported that oxidative stress caused by excessive training may compromise performance in athletes (Hattori et al., 2009; Tanskanen et al., 2010; Marin et al., 2013). Oxidative stress plays a critical role in overtraining syndrome in athletes (Tanskanen et al., 2010), which can be associated with fatigue, as well as lower muscle contractile function (Moopanar and Allen, 2005; Powers et al., 2011; Cheng et al., 2016), suggesting that oxidative stress is a target that needs to be controlled in athletes (Purvis et al., 2010; Pingitore et al., 2015). Previous studies have shown that supplementation with natural antioxidants is an efficient strategy to prevent oxidative stress in different kinds of athletes (Pingitore et al., 2015). For example, it has been demonstrated that supplementation with integral purple grape juice for 28 days improved running time-to-exhaustion in recreational runners as well as increasing antioxidant activity (Toscano et al., 2015).

In the present study, we demonstrated that 28 days of SC nectar was effective for reducing MDA and increasing TAC in young handball players, leading to protective effects against oxidative stress. Currently, there is a lack of studies investigating the effects of SC in humans. However, our findings are in agreement with a study conducted by Ulla et al. (2017), which investigated the effects of SC seed powder supplementation on oxidative stress in rats with high fat diet induced obesity. The findings showed that SC prevented oxidative stress by decreasing MDA levels and increasing activity of antioxidant enzymes (SOD, CAT, and GPX).

The improvements in redox homeostasis by SC supplementation may be explained by the high levels of phenolic compounds (Ayyanar and Subash-Babu, 2012), such as gallic acid and flavonoids (i.e., catechin, epicatechin, epigallocatechingallate, and epicatechingallate) as demonstrated in the present study, as these bioactive nutrients can generate antioxidant and anti-inflammatory activity as well as hypoglycemic effects (Meydani and Hasan, 2010; Kosuru et al., 2018). Studies have demonstrated that gallic acid, epicatechin, and epigallocatechingallate can activate a transcription factor denominated nuclear factor erythroid 2-related factor 2 (Nfr2), which in turn is translocated to the nucleus cell to synthesize antioxidant enzymes (Done and Traustadottir, 2016; Shin et al., 2016; Feng et al., 2017). We hypothesize that SC nectar supplementation increased TAC mediated by Nfr2 activation; however, future research is required to test this hypothesis.

Serum CK and LDH levels may be increased due to skeletal muscle damage as a consequence of intense training and are directly associated with training load (Horta et al., 2017). In addition, muscle damage can induce delayed-onset muscle soreness and performance loss (Peake and Neubauer, 2017). For this reason, persistently increased levels of serum CK are considered a biomarker of overtraining, often used for monitoring athletes in sport medicine (Brancaccio et al., 2007; Carfagno and Hendrix, 2014). We observed that SC supplementation influenced resting CK and LDH levels, with a significant reduction only in the SC group compared to baseline. These results may be explained by the influence of ROS production on the etiology of muscle damage through ion transport system oxidation (Kourie, 1998), as oxidative stress can lead to the release of muscle constituents to blood, such as CK and LDH (Armstrong, 1990; Taghiyar et al., 2013). Supporting this, previous studies have demonstrated that dietary antioxidant ingestion reduced biomarkers of muscle damage after fatiguing exercise (Taghiyar et al., 2013; Pereira Panza et al., 2015). Recently, Machado et al. (2018) demonstrated that green tea extract supplementation before an event of cumulative fatigue reduced muscle damage and oxidative stress in trained athletes. Thus, lower oxidative stress in the SC group may explain the reduction in serum CK and LDH levels, demonstrating that SC nectar supplementation could be an important strategy to maintain lower CK and LDH concentration during rest in young athletes.

Furthermore, the routine of athletes can induce changes in psychological response, such as mood disturbance, depression, difficulty in concentrating, and emotional instability, which are possible factors that explain performance loss during overtraining conditions (Purvis et al., 2010; Carfagno and Hendrix, 2014). In addition, increases in mood disturbance are associated with training load (Morgan et al., 1987), demonstrating that monitoring mood state may be a potential method of preventing performance reduction in athletes. It has been demonstrated that brain oxidative stress is associated with neurodegenerative and psychological disorders, contributing to mood disturbance (Salim, 2014, 2017). Our results showed that SC nectar supplementation improved psychological response with lower mood disturbance after 28 days of intervention. We hypothesize that the improvements in redox homeostasis through decreased MDA levels and increased TAC may be one potential mechanism that SC nectar supplementation reduced mood disturbance, although further studies are necessary to confirm the influence of natural antioxidants on psychological response in athletes and also in patients with psychological disorders. In addition, we observed significant correlations between changes in CK concentration and mood disturbance only for the SC group but not the placebo. Corroborating with our data, Hollander et al. (2016) found an association between serum CK and psychological disorders, suggesting that CK may be used as a potential biomarker of affective state. Thus, further studies are necessary to understand the relationship between CK levels and psychological response.

Finally, our results showed that despite the improvement in psychological parameters and redox homeostasis with SC nectar supplementation, we did not observe a significant difference between the placebo and SC groups for performance gains, as both groups presented increased vertical jump and anaerobic performance. These findings may be explained as the subjects are well trained and only 1 month of supplementation may be insufficient to verify significant improvement in performance. Therefore, we suggest further studies analyzing the effects of SC supplementation for more than 28 days on performance in different kinds of athletes and practitioners of physical activity.

Despite the importance of our data, some limitations need to be considered; the short-term of the intervention (28 days); the fact that data collection was performed during the pre-season and cannot be broken down into player positions to investigate the differences between positions, since there were a small number of athletes, mainly pivot position and physical activities in the remained hour of the day was not assessment. Also, we suggest further studies investigating the pharmacokinetics of the compound, once the effects could be of the last dose rather than an adaptation to the continuous SC supplementation.

## Conclusion

The present study suggests that SC nectar supplementation reduced biomarkers of oxidative stress and muscle damage, and improved psychological response in highly trained young handball players.

## Clinical Implications

The present study suggests that SC nectar supplementation can be used as a natural compound to help in exercise recovery and damage prevention in athletes. The results of this study may be applied by coaches and nutritionists could be an important non-pharmacological and a natural compound for minimize oxidative stress, muscle damage, and improve psychological response and maybe an interesting strategy to prevent overtraining syndrome in highly trained young athletes.

## Author Contributions

MdS, FR, and LC devised the study design, participated in the interpretation of data, and drafted the manuscript. FR, AS, AKB, and ACB arried out the data collection, participated in the interpretation of data, and assisted in the writing the manuscript. MeM, MdF, MdS, SR, AS, and RdM participated in the interpretation of data and drafted the manuscript. FR performed all statistical analysis, participated in the interpretation of data, and assisted in the writing of the manuscript. All authors read and approved the final manuscript.

## Conflict of Interest Statement

The authors declare that the research was conducted in the absence of any commercial or financial relationships that could be construed as a potential conflict of interest.

## Footnotes

1 www.randomizer.org

2 http://biostat.mc.vanderbilt.edu/wiki/Main/PowerSampleSize
